# Effects of English learning gains among college students on learning engagement: subjective wellbeing as a mediating variable and pro-environmental behavior as a moderating variable

**DOI:** 10.3389/fpsyg.2025.1688549

**Published:** 2026-01-21

**Authors:** Chengli Zhang, Ying Han, Aijun Yang

**Affiliations:** 1 Department of Foreign Languages, Xinzhou Normal Univerisity, Xinzhou, China; 2 Department of Tourism Management, Xinzhou Normal Univerisity, Xinzhou, China; 3 Public Foreign Language Department, Xinzhou Normal Univerisity, Xinzhou, China

**Keywords:** English learning engagement, learning gains, mediated moderating effects, pro-environmental behavior, subjective wellbeing

## Abstract

**Purpose:**

In today’s diverse social environment, college students often struggle to devote sufficient time to English learning, potentially affecting their academic performance, psychological wellbeing, social development, and career prospects. This study examined a moderated mediation model to explore how English learning gains influence learning engagement, with subjective wellbeing as a mediator and pro-environmental behaviors as a moderator.

**Methods:**

A total of 1,354 valid responses were collected from 1,500 students at an independent university in Shanxi Province, China. Data were analyzed using SPSS 27.0 and PROCESS 4.0, applying Pearson correlation and hierarchical linear regression analyses. The results revealed significant positive associations among pro-environmental behaviors, English learning gains, subjective wellbeing, and learning engagement.

**Results:**

English learning gains significantly predicted learning engagement, and subjective wellbeing mediated this relationship. Pro-environmental behaviors moderated the mediating pathway, strengthening the effects of both learning gains and subjective wellbeing on learning engagement.

**Conclusion:**

These findings provide empirical evidence on the mechanisms underlying college students’ English learning engagement and offer practical guidance for educational institutions to support student learning through wellbeing enhancement and pro-environmental initiatives.

## 1 Introduction

With globalization advancing, English learning has become a central component of higher education, shaping college students’ academic development and future career opportunities (Rahman and Singh, 2020). While researchers widely acknowledge the importance of learning engagement in language acquisition, student engagement in English learning varies considerably despite similar instructional settings. Understanding what drives students to sustain their effort and involvement therefore remains a critical concern.

A growing body of research suggests that learning gains—students’ perceived improvement in language knowledge and skills—are one of the strongest predictors of learning engagement (Daumiller et al., 2023; Pike et al., 2012). When students recognize progress, they are more willing to invest time and energy in learning. However, recent studies show that this relationship is not purely direct. Instead, subjective wellbeing plays a key psychological role: students who feel satisfied and emotionally positive about their learning experience are more likely to remain engaged (Pietarinen et al., 2014; Boulton et al., 2019). Thus, subjective wellbeing may function as an internal mechanism linking learning gains to engagement.

In higher education contexts, learning engagement has been widely recognized as a central determinant of students’ academic success and long-term development. However, what drives engagement and why certain students remain more engaged than others remains insufficiently understood. Although existing research confirms that learning gains can enhance motivation and engagement, the relationship is unlikely to be linear. Instead, it is shaped by students’ emotional experiences and individual behavioral patterns, making it essential to clarify the internal mechanisms that link these constructs.

Recent research in educational psychology highlights that learning engagement is influenced by both behavioral processes and affective experiences—the two core dimensions of student engagement identified in international scholarship (Fredricks et al., 2004; Skinner and Pitzer, 2012). From an affective perspective, emotions such as interest, enjoyment, and subjective wellbeing play a crucial role in sustaining engagement. Positive affect promotes greater persistence and deeper cognitive involvement, whereas negative emotions such as anxiety and stress undermine participation (Meyer and Turner, 2006). Subjective wellbeing (SWB), which reflects individuals’ cognitive and emotional evaluations of life, has gained increasing attention in learning research. Studies consistently show that SWB is positively associated with motivation, engagement, and academic effectiveness across diverse cultural contexts (Diener et al., 2023; Arockiam et al., 2012). Furthermore, improvements in students’ perceived learning gains can enhance their wellbeing, forming a mutually reinforcing cycle that strengthens engagement over time (Boulton et al., 2019). These findings suggest that SWB may operate as a key mediating mechanism through which learning gains translate into higher engagement.

From a behavioral perspective, students’ learning behaviors—including self-regulation, time management, and strategic learning—are strongly predictive of engagement levels. Effective behavioral strategies improve learning outcomes and increase the likelihood that students remain actively engaged in academic tasks (Khiat, 2019; Hickman et al., 2022). Poor learning habits, in contrast, are negatively associated with engagement (Li and Xue, 2023). Self-regulation and self-control, in particular, have been identified as robust predictors of engagement, partly through fostering constructive responses to academic challenges (Yang et al., 2024). These studies highlight the importance of integrating both emotional and behavioral pathways when examining how learning gains translate into engagement.

Although pro-environmental behavior (PEB) is traditionally situated within environmental psychology, emerging evidence suggests that PEB is associated with higher levels of subjective wellbeing and life satisfaction (Capaldi et al., 2014; Suárez-Varela et al., 2016). Engagement in PEB may strengthen students’ sense of purpose, self-efficacy, and responsibility—factors known to contribute to positive affective experiences. Given that SWB is linked to engagement, PEB may indirectly reinforce the emotional benefits of learning gains. However, the role of PEB within academic learning processes remains underexplored, and few studies have examined whether it strengthens the relationship between learning gains, SWB, and engagement. This gap is especially evident in language learning contexts, where PEB has rarely been conceptualized as an individual difference factor that may shape emotional and motivational outcomes.

To address these gaps, the present study investigates how English learning gains influence learning engagement, with a focus on subjective wellbeing as a mediator and pro-environmental behavior as a moderator. By integrating emotional and behavioral pathways within a single framework, this study proposes a moderated mediation model that clarifies the conditional processes through which learning gains enhance engagement. The following section defines the four core variables—learning gains, subjective wellbeing, learning engagement, and pro-environmental behavior—and develops the hypotheses that guide the research framework.

## 2 Theoretical basis and hypothesis

### 2.1 Relationship between learning gains and learning engagements

Learning gains refer to the knowledge, skills, competencies, and affective experiences learners acquire during the learning process (Rogaten and Rienties, 2021). In foreign language learning, they include language proficiency, intercultural communicative competence, and independent learning ability (Kolyada, 2021). Beyond academic performance, learning gains also involve students’ perceptions of progress, achievement, and self-efficacy (Hui et al., 2021). Factors influencing learning gains include learning strategies, motivation, and external support (Li et al., 2023). Limited exposure to authentic language environments in China can constrain English learners’ progress, highlighting the importance of effective learning strategies (Zhou, 2006; Xie, 2002).

Learning engagement is a multidimensional construct encompassing behavioral, affective, and cognitive components (Wang et al., 2019; Manavella et al., 2021). High engagement promotes motivation, strategy use, and persistence in language learning (Cheng, 2025). Conversely, limited real-world practice can reduce interest and lead to burnout, while extracurricular language activities reinforce learning (Sulis, 2023).

Social cognitive theory emphasizes that self-efficacy, strengthened by learning gains, enhances motivation and engagement (Bandura, 1997; Kenedi et al., 2023; Fredricks et al., 2004). Self-determination theory further highlights that intrinsic motivation fosters deeper engagement and academic improvement (Kanellopoulou and Giannakoulopoulos, 2020). A supportive learning environment and well-designed instructional strategies can enhance both learning gains and engagement (Nainggolan, 2024). Overall, learning gains and engagement interact dynamically and reinforce each other.

*H1*: Learning gains are positively related to learning engagement.

### 2.2 Mediating role of subjective wellbeing

Subjective wellbeing, first conceptualized by Diener, refers to individuals’ subjective evaluations of their quality of life, encompassing positive affect, negative affect, and life satisfaction (Diener, 1994). Subsequent research has expanded this concept to include self-actualization and psychological fulfillment, emphasizing its broader role in personal development (Kharytynskyi, 2022). Lyubomirsky et al. further proposed that subjective wellbeing shapes individuals’ cognitive processes, behaviors, and social interactions, thereby facilitating sustained personal growth (Lyubomirsky et al., 2005).

A growing body of research has demonstrated a close association between subjective wellbeing and academic outcomes, with higher levels of wellbeing linked to better academic performance and adaptive learning behaviors (Gilman and Huebner, 2006; Suldo et al., 2011). Emotional experiences, social support, and environmental adaptation have been identified as key antecedents of subjective wellbeing in educational contexts (Hidalgo-Fuentes et al., 2024). Recent studies further suggest that subjective wellbeing serves as an important psychological resource that enhances students’ learning gains (Abbasi and Lin, 2023). Conversely, negative emotions such as academic anxiety may undermine life satisfaction and hinder learning processes (Steinmayr et al., 2016). By alleviating academic stress and enhancing resilience, subjective wellbeing enables learners to maintain focus and persistence in academic tasks.

Accordingly, this study proposes the following hypothesis:

*H2a*: Subjective wellbeing is positively related to learning gains.

Subjective wellbeing also plays a critical role in promoting learning engagement. Learners with higher wellbeing tend to perceive their learning environment more positively, allocate greater cognitive and emotional resources to learning, and cope more effectively with academic challenges (Rüppel et al., 2015). From the perspective of positive psychology, positive emotional experiences broaden individuals’ cognitive resources and enhance self-efficacy, thereby fostering sustained engagement in learning activities (Fredrickson, 2001). Similarly, self-determination theory posits that wellbeing strengthens intrinsic motivation, increasing learners’ initiative and persistence (Deci and Ryan, 2000). Empirical evidence indicates that students with higher subjective wellbeing demonstrate greater concentration, more active classroom participation, and stronger willingness to engage in deep learning (Datu and King, 2018).

Based on these findings, the following hypothesis is proposed:

*H2b*: Subjective wellbeing is positively correlated with learning engagement.

More importantly, prior research suggests a reciprocal relationship between learning gains and subjective wellbeing. Learning gains derived from academic progress and personal development can enhance learners’ subjective wellbeing (Fang, 2022), which in turn promotes sustained learning engagement (Lai, 2024). Positive learning experiences—such as improvements in language proficiency or cognitive skills—foster feelings of accomplishment and satisfaction, thereby strengthening intrinsic motivation and willingness to invest continued effort in learning (Elsan et al., 2022). As a result, subjective wellbeing functions as a key psychological mechanism linking learning gains to learning engagement (Kahu and Nelson, 2018).

Therefore, this study proposes the following hypothesis:

*H2*: Subjective wellbeing mediates the relationship between learning gains and learning engagement.

### 2.3 Moderators of pro-environmental behavior

Previous research has shown that engagement in pro-environmental behavior is positively associated with individuals’ subjective wellbeing (Suárez-Varela et al., 2016). From the perspective of the Happiness–Altruism Cycle, sustained prosocial behaviors—such as environmentally responsible actions—may foster a positive self-concept, enhanced self-efficacy, and a sense of purpose, which together contribute to higher levels of wellbeing (Wu et al., 2023). Empirical evidence further suggests that participation in environmental volunteering is associated with greater life satisfaction and emotional wellbeing compared to non-participation (Kragh et al., 2016).

Higher subjective wellbeing, in turn, has been linked to increased investment in learning activities, including greater persistence, effort, and engagement (Datu and King, 2018). From a psychological capital perspective, individuals who regularly engage in value-driven behaviors, such as pro-environmental actions, may exhibit higher levels of hope, resilience, and confidence (Priya and Thenmozhi, 2021). These psychological resources are not domain-specific and may generalize to academic contexts, thereby supporting sustained learning engagement (Chen, 2020). In addition, pro-environmental behavior is often associated with a stronger sense of responsibility and goal orientation (Yang, 2024), which may coexist with higher levels of active participation in learning activities (He et al., 2021).

In summary, the present study proposes the following hypotheses:

*H3a*: Pro-environmental behavior is positively associated with subjective wellbeing.

*H3b*: Pro-environmental behavior is positively associated with learning engagement.

*H3*: Pro-environmental behavior moderates the second stage of the pathway linking learning gains to learning engagement via subjective wellbeing.

Prior research suggests that individuals’ engagement in pro-environmental behavior is influenced by multiple psychological and contextual factors, including environmental awareness, affective appraisal, self-determined motivation, and social norms (Gifford and Nilsson, 2014). For example, individuals with higher levels of environmental awareness are more likely to engage in protective actions due to heightened concern for environmental issues (Suárez-Varela et al., 2016). Social norms also play a critical role, as observing pro-environmental behavior within one’s social environment increases the likelihood of adopting similar behaviors (Perry et al., 2021). Together, these factors shape individuals’ engagement in pro-environmental behavior across diverse social and educational contexts.

Although empirical research directly linking learning gains to pro-environmental behavior remains limited, existing evidence suggests several plausible connections. Positive learning experiences may enhance environmental awareness, while interactions with environmentally conscious peers can foster more responsible behavioral orientations (Melin, 2012). Moreover, learning gains may indirectly promote pro-environmental behavior by strengthening individuals’ self-efficacy and sense of purpose. When learners experience achievement and personal growth through learning, they may be more inclined to persist in goal-oriented and socially responsible behaviors, including environmental protection efforts (Priya and Thenmozhi, 2021).

Importantly, pro-environmental behavior is shaped not only by cognitive factors but also by emotional empathy and a broader sense of social responsibility (Zelenski and Desrochers, 2021). Previous studies have examined various antecedents of such behaviors, including the role of environmental education in promoting ecological action (Ma et al., 2023), the relationship between green consumption and environmental attitudes (Sun et al., 2019), and the influence of environmental concern on sustainable practices (Sulphey and Faisal, 2021).

Based on this body of literature, the present study further proposes:

*H4a*: Pro-environmental behavior is positively associated with learning gains.

*H4*: Pro-environmental behavior moderates the relationship between learning gains and learning engagement.

Importantly, the present study does not assume that pro-environmental behavior is uniquely positioned to influence academic engagement, nor does it claim causal primacy over other forms of prosocial behavior (e.g., volunteering or community service). Rather, pro-environmental behavior is treated as one illustrative example of a sustained, value-oriented prosocial orientation. Its inclusion is exploratory in nature and aims to examine whether such orientations are associated with variations in learning engagement processes.

## 3 Materials and methods

### 3.1 Participants and procedures

This study was conducted at a full-time undergraduate normal university in Shanxi Province, which enrolls approximately 20,000 students. The university English program, a major course, is compulsory for first-year students. Therefore, the questionnaire targeted first-year students. At the end of the English course, 1,500 students were randomly selected, and the validity of the questionnaire was assessed after data collection, resulting in 1,354 valid responses.

### 3.2 Data collection

Data were collected via a questionnaire distributed from September 26 to December 24, 2023. Students accessed the questionnaire by scanning a QR code during intermissions of their college English and English major courses, as organized by their instructors. Scanning the QR code directed students to the questionnaire, where they could answer and submit their responses. (A QR code, or Quick Response code, is an efficient tool for information transfer, widely used in China for accessing web content, financial transactions, identity verification, and information sharing).

Before scanning the QR code, teachers explained the purpose of the study in detail to students. In addition, participants were informed that they could choose to participate voluntarily and could withdraw at any time without any penalty. Although the participants were adults, parents were also informed to ensure transparency and ethical oversight, particularly for first-year students who may be living away from home for the first time. These procedures aimed to ensure voluntary participation while maintaining ethical standards.

### 3.3 Measures

The questionnaire consisted of five sections comprising a total of 79 items: demographic information, learning engagement scale, pro-environmental behavior scale, learning gain scale, and subjective wellbeing scale. Demographic data encompassed gender, home address, and major, which were collected for descriptive purposes but were not included as covariates in the analyses due to limited variance among first-year students, and thus their explanatory power in the structural model was considered minimal. The original scales were designed in English, so reverse translation was employed to ensure accuracy: one researcher translated the questionnaire into Chinese, another researcher re-translated it into English, while a third researcher analyzed the two versions and calibrated the scales to rectify discrepancies. To improve the quality of translation, this study adopted the back-translation method (Brislin, 1970). Hence, the initial researcher translated the English text into Chinese, followed by the second researcher who translated the Chinese rendition back into English. Subsequently, the third researcher compared the scales of the original, translated, and back-translated versions to evaluate the accuracy of the translation. Before finalizing the questionnaire, the translation was rectified and enhanced, which ensured the equivalence of the scale.

#### 3.3.1 Learning gains scale

The questionnaire included personal information (gender and grade), an English classroom learning engagement scale, and a learning gain satisfaction scale. The learning engagement scale was based on state descriptions of language learning engagement by scholars such as Philp and Duchesne (2016) and aligned with the dimensions established in the Multidimensional Evaluation Scale of College Students’ Learning Engagement in the English Classroom (Ren, 2022). Drawing on Zilvinskis et al. (2017) and other self-reported learning gain evaluation dimensions, the scale was revised multiple times.

The Learning Gain Satisfaction Scale comprised sixteen items evaluated on a scale with five stars ranging from 1 (“not at all consistent”) to 5 (“fully consistent”). The Academic Development subscale included nine items assessing language proficiency (e.g., “Learning in the English classroom helps me improve my reading ability”) and learning ability (e.g., “Learning in the English classroom helps me improve my ability to investigate based on the information I have already obtained”). The Mental Development subscale contained seven items measuring applied competence (e.g., “Learning in the English classroom helps me to improve my ability to solve practical problems”) and general literacy (e.g., “Learning in the English classroom helps me to establish a correct outlook on the world, life, and values”). The scale demonstrated a Cronbach’s alpha coefficient of 0.974.

#### 3.3.2 Learning engagement scale

The learning engagement scale adopted the multidimensional evaluation scale of English classroom learning engagement constructed by Ren (2022) for college students in the Chinese foreign language teaching context. It includes three dimensions: “behavioral input,” “affective input,” and “cognitive input.” The Behavioral Input Scale includes nine items measuring teacher-student interaction (e.g., “I actively answer the teacher’s questions in the English classroom”), individual effort (e.g., “I seriously think about the difficulties I encounter in the English classroom”), and peer interaction (e.g., “I cooperate with other students to complete learning tasks in English class”). The Emotional Engagement component comprises 10 items that assess pleasant feelings. (e.g., “Learning new things in English class is enjoyable for me”), interest-driven motivation (e.g., “I am interested in the content and topics of English class”), and group belonging (e.g., “I like the learning atmosphere in the English classroom”).

The Cognitive Engagement subscale includes 11 items assessing self-planning (e.g., “I take the initiative to make a learning plan for English class”), deeper learning (e.g., “I emphasize a deeper understanding of what I have learned in English class”), and self-monitoring (e.g., “I take the initiative to regulate negative emotions that arise in English class”). Every item was evaluated using a five-point Likert scale. with responses ranging from “almost never” to “always.” The scale demonstrated a Cronbach’s alpha coefficient of 0.971.

#### 3.3.3 Subjective wellbeing scale

The Subjective Wellbeing Scale, proposed by Diener (1984), is an individual’s overall evaluation of life quality based on personal criteria. It contains two main dimensions: affective and cognitive. Alongside the Psychological Wellbeing Scale (PWBS), it is among the most widely used instruments for measuring wellbeing in China. Chinese scholar Xing (2002) adapted the Subjective Wellbeing Scale into a 20-item Chinese version. Participants utilized a five-point scale, with “1” signifying “strongly disagree” and “5” denoting “strongly agree.” The Cronbach’s alpha coefficient for the Subjective Wellbeing Scale in this study was 0.817.

#### 3.3.4 Pro-environmental behavior scale

Pro-environmental behavior was assessed using several established constructs. Place attachment was measured using six items related to place identity and place emotion, based on Williams and Vaske (2003) and Kyle et al. (2004). Subjective norms were assessed using three semantic differential items recommended by Ajzen (1991) and Fielding et al. (2008). Perceived behavioral efficacy was measured with four items adapted from studies on pro-social and pro-environmental behavior (Steg and de Groot, 2010; Stern et al., 1999). The willingness for environmentally responsible behavior (ERB) was assessed using 10 questions derived from the scales of Halpenny (2006) and Ramkissoon et al. (2013), adapted for the Chinese setting. All questions were evaluated using a five-point Likert scale (1 = strongly disagree, 5 = strongly agree). The Cronbach’s alpha coefficient for pro-environmental behavior in this study was 0.913.

### 3.4 Data analysis

The data were processed and analyzed using SPSS 27.0. Demographic variables, including gender, major, and home address, were collected to describe the sample characteristics. Preliminary examinations indicated limited variability in these variables (e.g., a high proportion of female students and relatively homogeneous academic backgrounds among first-year students); therefore, they were not included as control variables in the main analyses due to their limited explanatory power for the structural model. Meanwhile, several procedures were implemented during the study design and data collection to mitigate self-report bias and common method variance. For example, prior to questionnaire administration, participants were informed of the anonymity and confidentiality of the survey and that there were no “right” or “wrong” answers, in order to reduce social desirability bias. All measurement instruments were adapted from well-established scales and translated using a back-translation procedure to ensure content accuracy and validity.

At the statistical level, Harman’s single-factor test was conducted on the 76 items representing the four core variables to assess common method variance. The results showed that 11 factors had eigenvalues greater than 1, accounting for 67.634% of the total variance. Importantly, the first factor explained only 34.177% of the variance, which is below the critical threshold of 40% (Zhou and Long, 2004), indicating that common method bias was unlikely to pose a serious threat to the study results. After confirming that common method variance was not a major concern, descriptive statistics, correlation analyses, and hypothesis testing were conducted. Descriptive statistics were used to examine the central tendency and dispersion of the data, while Pearson correlation analyses were employed to assess the relationships among the independent, mediating, dependent, and moderating variables. Finally, the study employed the PROCESS macro (version 4.0) for SPSS to test mediation and moderated mediation models. This macro, developed by Hayes (2017), is specifically designed for examining mediation, moderation, and their integration using regression-based path analysis.

## 4 Results

### 4.1 Descriptive statistics and correlations analyses

The study analyzed the mean values, standard deviations, and correlations among learning gain, learning engagement, subjective wellbeing, and pro-environmental behavior using descriptive statistics and Pearson’s correlation coefficients in SPSS 27.0. Table 1 presents the descriptive statistics and correlation matrix. Learning gain exhibited a positive correlation with pro-environmental behavior (*r* = 0.375, *p* < 0.01), learning engagement (*r* = 0.670, *p* < 0.01), and subjective wellbeing (*r* = 0.126, *p* < 0.01). Pro-environmental behavior was positively associated with learning engagement (*r* = 0.413, *p* < 0.01) and subjective wellbeing (*r* = 0.083, *p* < 0.01). Learning engagement was also significantly correlated with subjective wellbeing (*r* = 0.141, *p* < 0.01). These correlations suggest that students who report higher learning gains tend to demonstrate stronger learning engagement and higher levels of pro-environmental behavior, providing preliminary support for the hypothesized relationships.

**TABLE 1:** Descriptive statistics and correlation.

Variables	Mean	Standard deviation	Number of cases	Pro-environmental behavior	Learning gains	Learning engagement	Subjective wellbeing
Pro-environmental behavior	3.3395	0.64927	1354	1			
Learning gains	3.641	0.75638	1354	0.375**	1		
Learning engagement	3.1105	0.69739	1354	0.413**	0.670**	1	
Subjective wellbeing	2.9044	0.48154	1354	0.083**	0.126**	0.141**	1

**p < 0.01.

### 4.2 Mediation analysis

To examine the mediating role of subjective wellbeing, Model 4 of the PROCESS macro was employed. Learning gain was specified as the independent variable, learning engagement as the dependent variable, and subjective wellbeing as the mediator. As shown in Table 2, learning gain significantly predicted learning engagement (β = 0.611, SE = 0.019, *p* < 0.001), indicating a strong direct association. Learning gain also positively predicted subjective wellbeing (β = 0.080, SE = 0.017, *p* < 0.001). Subjective wellbeing, in turn, positively predicted learning engagement (β = 0.083, SE = 0.029, *p* < 0.001).

**TABLE 2:** Mediating role of subjective wellbeing.

Predictors	Subjective wellbeing					Learning engagement				
	β	SE	*t*	95%CI		β	SE	*t*	95%CI	
				LLCI	ULCI				LLCI	ULCI
Learning gains	0.08	0.0172	4.6584***	0.0463	0.1137	0.611	0.0187	32.656***	0.5744	0.6478
Subjective wellbeing						0.083	0.0294	2.8353***	0.0257	0.141
R-squ	0.0158					0.4522				
*F*	21.7006					557.6749				

***p < 0.001. Analyses conducted by PROCESS Model 4, *N* = 1,354. Gender is dummy coded (1, female, 2, male).

Bootstrapping with 5,000 samples was conducted to estimate confidence intervals for the direct and indirect effects. The 95% confidence intervals for both effects did not include zero, indicating statistical significance. The indirect effect of learning gain on learning engagement via subjective wellbeing was β = 0.007, accounting for only 1.1% of the total effect, while the direct effect accounted for 98.9% (Table 3).

**TABLE 3:** Total, direct, and indirect effects associated with variables.

Effect type	Effect size	Boot SE	95%CI		Relative effect size
			LLCI	ULCI	
Total effect	0.618	0.0186	0.5813	0.6543	
Direct effect	0.611	0.0187	0.5744	0.6478	98.9%
Indirect effect	0.007	0.0035	0.0007	0.0145	1.1%

Analyses conducted by PROCESS Model 4, *N* = 1,354.

### 4.3 Moderated mediation analysis

**FIGURE 1:**
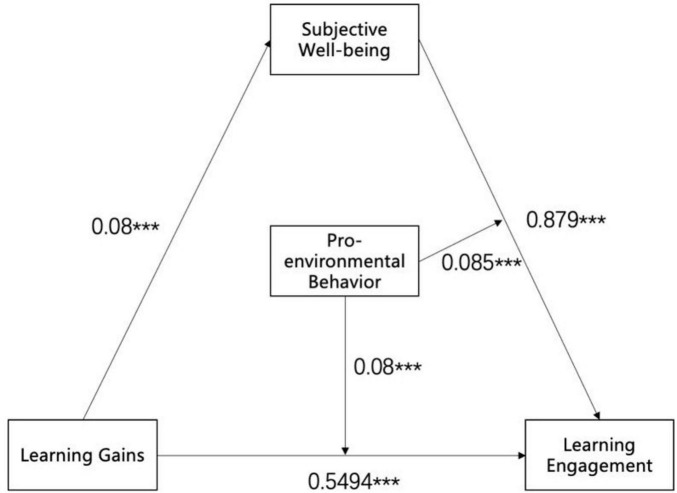
English learning gains among college students on learning engagement: subjective wellbeing as a mediating variable and pro-environmental behavior as a moderating variable (Model 15). ***p < 0.001.

Given the modest mediating effect of subjective wellbeing, pro-environmental behavior was introduced as a moderating variable to further explore boundary conditions of the model. PROCESS Model 15 was applied to test the moderated mediation framework. As shown in Table 4, the interaction between learning gain and pro-environmental behavior significantly predicted learning engagement [β = 0.080, *p* < 0.001, 95% CI (0.0362, 0.1239)]. Similarly, the interaction between subjective wellbeing and pro-environmental behavior was significant (β = 0.085, *p* < 0.001, 95% CI [0.0173, 0.1527]). However, the interaction coefficients were small in magnitude, suggesting that pro-environmental behavior functions as a weak contextual moderator whose influence is detectable at the statistical level (Figure 1).

**TABLE 4:** Moderating effects of pro-environmental behaviors.

Predictors	Learning Gains					Learning engagement				
	β	SE	*t*	95%CI		β	SE	*t*	95%CI	
				LLCI	ULCI				LLCI	ULCI
Learning gains	0.08	0.0172	4.6584***	0.0463	0.1137	0.549	0.0194	28.2706***	0.5113	0.5876
Subjective wellbeing						0.088	0.0286	3.0797***	0.0319	0.1439
Pro-environmental behaviors						0.207	0.0229	9.0015***	0.1615	0.2515
Learning gains × Pro-environmental behaviors						0.08	0.0224	3.5787***	0.0362	0.1239
Subjective wellbeing × Pro-environmental Behaviors						0.085	0.0345	2.4615***	0.0173	0.1527
R-squ	0.0158					0.4910				
*F*	21.7006					260.0256				

Analyses conducted using PROCESS model 15 with *N* = 1,354.

***p < 0.001.

**FIGURE 2:**
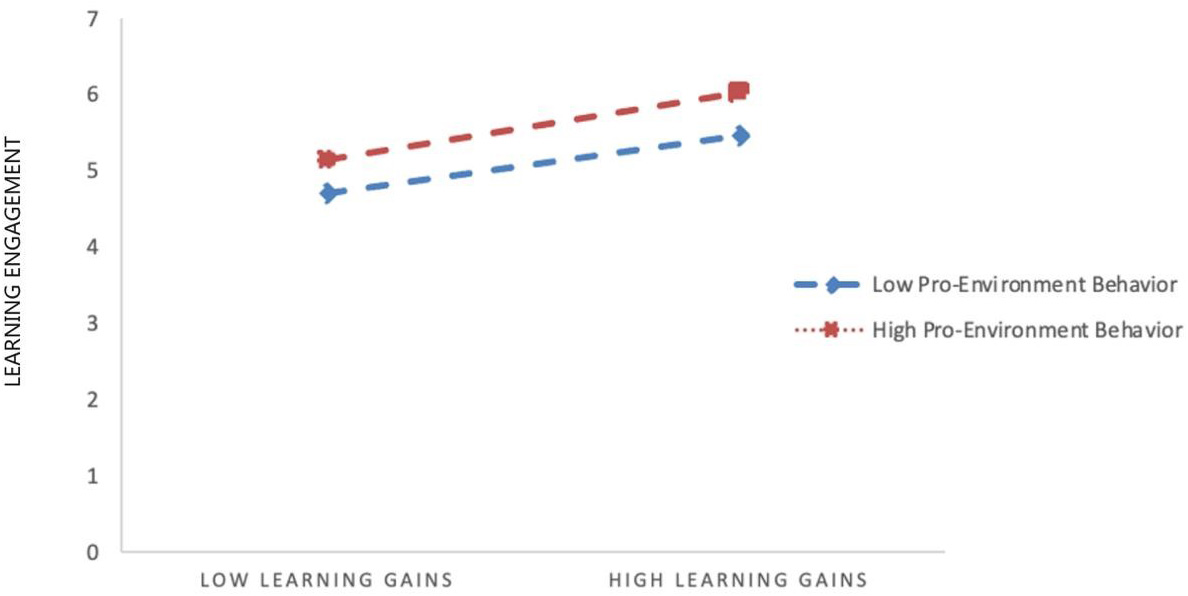
Moderating effects of pro-environmental behaviors in learning gains and learning engagement.

**FIGURE 3:**
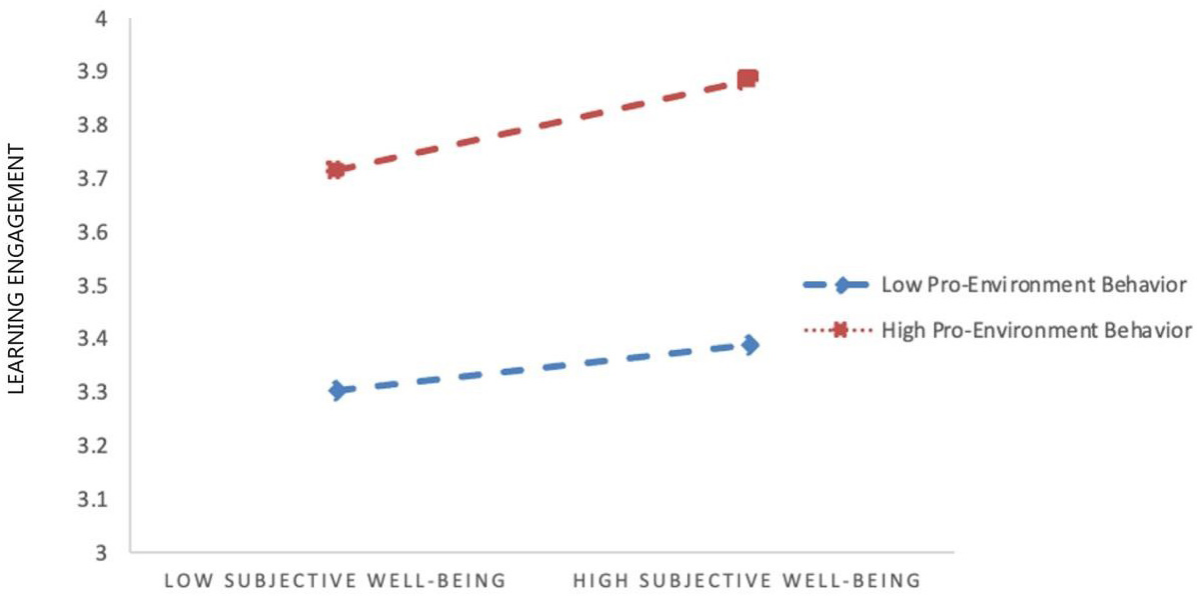
Moderating effect of pro-environmental behavior in the second half of the learning gains-subjective wellbeing-learning engagement pathway.

To illustrate these interaction effects, simple slope analyses were conducted by categorizing participants into low (M − 1 SD) and high (M + 1 SD) pro-environmental behavior groups. The results showed that the 95% confidence intervals did not include zero (Table 5). Figures 2, 3 depict these interaction patterns, indicating that higher levels of pro-environmental behavior slightly strengthen the associations between learning gain and learning engagement, as well as between subjective wellbeing and learning engagement.

**TABLE 5:** Moderating effects of pro-environmental behaviors.

Outcome variable	Variables			95%CI	
	Moderator level	Effect	SE	LLCI	ULCI
Learning engagement	Low (M-1SD)	0.515	0.0216	0.4725	0.5574
	Medium (M)	0.5441	0.0195	0.5059	0.5823
	High (M+1SD)	0.6023	0.0245	0.5543	0.6503
	Low (M-1SD)	0.0513	0.0318	−0.011	0.1137
Subjective wellbeing	Medium (M)	0.0822	0.0286	0.0262	0.1383
	High (M+1SD)	0.144	0.0371	0.0713	0.2168

Bootstrap sample size = 5,000. Low, 1 SD below the mean. High, 1 SD above the mean.

## 5 Discussion

First, the findings of this study indicate that learning gains are strongly associated with learning engagement. This result is consistent with Hypothesis H1 and suggests that students’ perceived gains in knowledge and skills are closely linked to higher levels of engagement in learning (Pike et al., 2012). Learning gains encompass not only knowledge acquisition but also skill development and personal growth (Rogaten and Rienties, 2021). When students perceive progress and improvement during the learning process, they are more likely to experience a sense of accomplishment, which is associated with greater motivation and engagement (Fredricks et al., 2004). These positive learning experiences may help sustain students’ involvement in academic activities and are consistently related to higher levels of learning engagement (Kesehatan et al., 2024).

Second, the results indicate that subjective wellbeing statistically mediates the relationship between learning gains and learning engagement; however, the magnitude of this mediation effect is very small. Although the indirect pathway reached statistical significance, it accounted for only a limited proportion of the total effect, suggesting that subjective wellbeing should not be interpreted as a strong or primary mechanism linking learning gains to learning engagement. Learning gains involve not only academic progress but also aspects of personal growth and self-development (Rogaten and Rienties, 2021). Through educational experiences, students may develop a greater sense of competence, confidence, and appreciation for learning, which can be associated with positive emotional states. Prior research has shown that perceived growth, such as improvements in critical thinking, is related to higher engagement (Rogaten and Rienties, 2021), and that subjective wellbeing may accompany such positive learning experiences (Elsan et al., 2022). However, in the present study, subjective wellbeing appears to function as a weak and secondary pathway rather than a substantive mediator. This suggests that while emotional wellbeing may coexist with learning gains, it plays a relatively minor role in explaining students’ learning engagement compared to the direct association between learning gains and engagement.

Third, the findings indicate that pro-environmental behavior is positively associated with both learning gains and learning engagement, which is consistent with Hypotheses H3a and H3b at the correlational level. Engagement in pro-environmental activities has been linked to greater environmental awareness, a sense of responsibility, and personal meaning, which may coexist with higher levels of academic involvement (Dasi et al., 2019). Such behaviors can strengthen students’ identification with pro-social and environmental values, contributing to a broader sense of social responsibility and collective orientation (Yang, 2024). These value orientations may, in turn, be associated with greater motivation and participation in learning activities (He et al., 2021). However, rather than implying direct or causal effects, the present findings suggest that pro-environmental behavior and learning-related outcomes tend to co-occur. Students who are more inclined to engage in socially and environmentally responsible behaviors may also be more likely to invest effort in their academic learning. Prior studies have shown that engagement in meaningful, value-driven activities is associated with higher subjective wellbeing and academic engagement (Zawadzki et al., 2020; Zimmerman, 2000). Importantly, although pro-environmental behavior was examined as a moderator in the model, the interaction effects involving subjective wellbeing and learning engagement were small in magnitude. Therefore, pro-environmental behavior should be interpreted as a contextual characteristic that is modestly related to learning engagement, rather than as a strong moderating mechanism shaping the relationship between subjective wellbeing and learning engagement.

Fourth, the results indicate that pro-environmental behavior modestly moderates the association between learning gains and learning engagement. Although the interaction effect reached statistical significance, its magnitude was small, suggesting that the moderating role of pro-environmental behavior should be interpreted cautiously rather than as a strong or decisive influence. Prior research has shown positive associations among pro-environmental behavior, academic performance, and students’ sense of responsibility (Dasi et al., 2019). Participation in environmental activities may contribute to the development of responsibility toward environmental stewardship (Yang, 2024), which can be associated with greater academic commitment and motivation (He et al., 2021). However, in the present study, pro-environmental behavior appears to function as a contextual characteristic that slightly shapes how learning gains relate to learning engagement, rather than as a mechanism that substantially amplifies this relationship.

## 6 Implications, limitations, and future directions

This study has three limitations. First, it adopts a cross-sectional design, which captures English learning engagement at a single point in time and therefore limits the inference of causal relationships and their dynamic changes. Future research employing longitudinal or experimental designs is needed to more systematically examine the causal mechanisms among learning gains, subjective wellbeing, and learning engagement.

Second, although the sample size was relatively large (*N* = 1,354) and participants were randomly selected from universities in Shanxi Province, China, the use of a single regional sample limits the generalizability of the findings. Given that educational systems, learning cultures, and sustainability-oriented values vary across countries and regions, future studies should replicate the proposed model in different cultural and educational contexts to enhance its cross-cultural applicability.

Third, although the mediating effect of subjective wellbeing between learning gains and learning engagement reached statistical significance, its effect size was small and its practical explanatory power was limited. This suggests that subjective wellbeing is more likely to function as a secondary or contextual psychological factor rather than a core explanatory mechanism. Similarly, the moderating effect of pro-environmental behavior was modest, indicating that it plays a contextual rather than decisive role in the relationship between learning gains and learning engagement.

In addition, beyond sampling and design constraints, these findings may also be influenced by potential measurement bias, omitted variables, and common method variance inherent in self-report data. Taken together, these limitations underscore the importance of interpreting statistical significance with caution and highlight the need for future research to adopt more rigorous measurement strategies and explore alternative mediating and moderating variables with greater explanatory potential.

Despite these limitations, the study offers several implications for teaching practice and educational policy. At the pedagogical level, the robust association between learning gains and learning engagement highlights the importance of promoting meaningful learning progress, skill development, and reflective learning experiences. At the policy level, the findings suggest that policies aimed at improving learning quality may enhance student engagement, even when the contribution of indirect psychological pathways is limited.

Within the context of global and sustainable education, the results indicate that integrating environmental responsibility and learner-centered pedagogical approaches into higher education may provide complementary support for learning engagement. However, given the relatively small effect sizes observed, these implications should be viewed as context-dependent and complementary rather than as universally applicable intervention strategies. Future cross-national comparative research is needed to further examine the role of sustainability-oriented educational practices across different cultural contexts.

## Data Availability

The original contributions presented in this study are included in this article/supplementary material, further inquiries can be directed to the corresponding author.

## References

[B1] Abbasi N. Lin L. (2023). Subjective well-being and school functioning among high school students. *J. Prof. Appl. Psychol.* 4 491–499. 10.52053/jpap.v4i4.225

[B2] Ajzen I. (1991). The theory of planned behavior. *Organ. Behav. Hum. Decis. Process.* 50 179–211. 10.1016/0749-5978(91)90020-T

[B3] Arockiam L. Charles S. Jayanthi M. Mercy A. (2012). An impact of emotional happiness in students learning environment. *CIIT Int. J. Data Min. Knowl. Eng.* 4 237–241.

[B4] Bandura A. (1997). The anatomy of stages of change. *Am. J. Health Promot.* 12 8–10. 10.4278/0890-1171-12.1.8 10170438

[B5] Boulton C. Hughes E. Kent C. Smith J. Williams H. (2019). Student engagement and wellbeing over time at a higher education institution. *PLoS One* 14:e0225770. 10.1371/journal.pone.0225770 31774878 PMC6881016

[B6] Brislin R. W. (1970). Back-translation for cross-cultural research. *J. Cross Cult. Psychol.* 1 185–216. 10.1177/135910457000100301

[B7] Capaldi C. A. Dopko R. L. Zelenski J. M. (2014). The relationship between nature connectedness and happiness: A meta-analysis. *Front. Psychol.* 5:976. 10.3389/fpsyg.2014.00976 25249992 PMC4157607

[B8] Chen Y. (2020). Correlation between self-efficacy and English performance. *Int. J. Emerg. Technol. Learn.* 15 223–234. 10.3991/ijet.v15i08.13697

[B9] Cheng W. J. (2025). The impact of vocational undergraduate students’ English learning motivation on academic performance. *University* 5 181–184.

[B10] Dasi A. Miarsyah M. Rusdi R. (2019). The relationship between personal responsibility and pro-environmental intention in high schools students. *J. Pendidikan Biol. Indones.* 5 17–22. 10.22219/jpbi.v5i1.7117

[B11] Datu J. King R. (2018). Subjective well-being is reciprocally associated with academic engagement: A two-wave longitudinal study. *J. Sch. Psychol.* 69 100–110. 10.1016/j.jsp.2018.05.007 30558746

[B12] Daumiller M. Rinas R. Dresel M. (2023). Relevance of students’ goals for learning engagement and knowledge gains in an online learning course. *Behav. Sci.* 13:161. 10.3390/bs13020161 36829390 PMC9952138

[B13] Deci E. L. Ryan R. M. (2000). The “what” and “why” of goal pursuits: Human needs and the self-determination of behavior. *Psychol. Inq.* 11 227–268. 10.1207/S15327965PLI1104_01

[B14] Diener E. (1984). Subjective well-being. *Psychol. Bull.* 95 542–575. 10.1037/0033-2909.95.3.542 6399758

[B15] Diener E. (1994). Assessing subjective well-being: Progress and opportunities. *Soc. Indic. Res.* 31 103–157. 10.1007/BF01207052

[B16] Diener E. Oishi S. Lucas R. E. (2003). Personality, culture, and subjective well-being: Emotional and cognitive evaluations of life. *Annu. Rev. Psychol.* 54, 403–425. 10.1146/annurev.psych.54.101601.145056 12172000

[B17] Elsan B. Sadeghoghli H. Rouhi A. (2022). Motivonia: Wellbeing and well-willing, two factors of English language learners’ motivated behaviour. *Soc. Welf.* 22 195–233. 10.32598/refahj.22.86.3566.1

[B18] Fang B. (2022). The impact mechanism of self-efficacy on learning gains among vocational college students: The mediating role of social support. *Vocat. Tech. Educ.* 43 47–52.

[B19] Fielding K. S. Terry D. J. Masser B. M. Hogg M. A. (2008). Integrating social identity theory and the theory of planned behavior to explain decisions to engage in sustainable agricultural practices. *Br. J. Soc. Psychol.* 47 23–48. 10.1348/014466607X206792 17535461

[B20] Fredricks J. A. Blumenfeld P. C. Paris A. H. (2004). School engagement: Potential of the concept, state of the evidence. *Rev. Educ. Res.* 74 59–109. 10.3102/00346543074001059 38293548

[B21] Fredrickson B. L. (2001). The role of positive emotions in positive psychology: The broaden-and-build theory of positive emotions. *Am. Psychol.* 56 218–226. 10.1037/0003-066X.56.3.218 11315248 PMC3122271

[B22] Gifford R. Nilsson A. (2014). Personal and social factors that influence pro-environmental concern and behaviour: A review. *Int. J. Psychol.* 49 141–157. 10.1002/ijop.12034 24821503

[B23] Gilman R. Huebner E. S. (2006). Characteristics of adolescents who report very high life satisfaction. *J. Youth Adolesc.* 35 293–301. 10.1007/s10964-006-9036-7

[B24] Halpenny E. A. (2006). *Environmental behavior, place attachment and park visitation: A case study of visitors to Point Pele National Park.* Waterloo: University of Waterloo.

[B25] Hayes A. F. (2017). *Introduction to mediation, moderation, and conditional process analysis: A regression-based approach.* New York, NY: Guilford Press.

[B26] He J. Wang Y. N. Zhuang M. K. Cheng H. Q. (2021). Factors influencing learning engagement of college freshmen and counseling strategies. *Educ. Acad. Mon.* 8, 57–64.

[B27] Hickman D. Glass W. West B. (2022). “Voices of educators,” in *Handbook of research on active learning and student engagement in higher education*, ed. Keengwe J. (Hershey, PA: IGI Global), 153–173. 10.4018/978-1-7998-9564-0.ch008

[B28] Hidalgo-Fuentes S. Martínez-Álvarez I. Sospedra-Baeza M. Martí-Vilar M. Merino-Soto C. Toledano-Toledano F. (2024). Emotional intelligence and perceived social support: Its relationship with subjective well-being. *Healthcare* 12:634. 10.3390/healthcare12060634 38540598 PMC10970432

[B29] Hui L. Hui X. Gu Q. (2021). Measurement of learning gains in higher education: An international comparative perspective. *Mod. Educ. Sci.* 1 149–156.

[B30] Kahu E. R. Nelson K. (2018). Student engagement in the educational interface: Understanding the mechanisms of student success. *High. Educ. Res. Dev.* 37 58–71. 10.1080/07294360.2017.1344197

[B31] Kanellopoulou C. Giannakoulopoulos A. (2020). Engage and conquer: An online empirical approach into whether intrinsic or extrinsic motivation leads to more enhanced students’ engagement. *Creat. Educ.* 11 143–165. 10.4236/ce.2020.112011

[B32] Kenedi G. Khoshkonesh A. Sharifi T. (2023). The relationship between academic self-efficacy and cognitive learning style (innovative or evasive) of adolescents during quarantine. *Razi J. Med. Sci.* 30 1–10. 10.47176/rjms.30.105 36128292

[B33] Kesehatan F. Masyarakat U. P. Makpal A. Yuspin S. Reypaty G. Kadore T. (2024). *The relationship of self-efficacy and self-adaptation to academic performance of nursing students.* Sulawesi Tengah: Media Publikasi Promosi Kesehatan Indonesia (MPPKI). 10.56338/mppki.v7i8.5644

[B34] Kharytynskyi A. (2022). Psychological content of the concept of subjective personal well-being. *Organ. Psychol. Econ. Psychol.* 3 149–159. 10.31108/2.2022.3.27.15

[B35] Khiat H. (2019). Using automated time management enablers to improve self-regulated learning. *Act. Learn. High. Educ.* 23 3–15. 10.1177/1469787419866304

[B36] Kolyada N. (2021). The importance of developing intercultural communication skills in teaching foreign languages. *Trends Dev. Sci. Educ.* 76 95–98. 10.18411/lj-08-2021-100

[B37] Kragh G. Stafford R. Curtin S. Díaz A. (2016). Environmental volunteer well-being: Managers’ perception and actual well-being of volunteers. *F1000Res.* 5:2679. 10.12688/f1000research.10016.1 28184285 PMC5288684

[B38] Kyle G. T. Mowen A. J. Tarrant M. (2004). Linking place preferences with place meaning: An examination of the relationship between place motivation and place attachment. *J. Environ. Psychol.* 24 439–454. 10.1016/j.jenvp.2004.11.001

[B39] Lai J. (2024). The relationship between college students’ subjective well-being and learning engagement: The mediating role of professional commitment. *J. Yanan Vocat. Tech. Coll.* 38 1–32. 10.13775/j.cnki.cn61-1472/g4.2024.04.004

[B40] Li J. Xue E. (2023). Dynamic interaction between student learning behaviour and learning environment: Meta-analysis of student engagement and its influencing factors. *Behav. Sci.* 13:59. 10.3390/bs13010059 36661631 PMC9855184

[B41] Li X. Liu W. Hu K. (2023). Learning motivation and environmental support: How first-generation college students achieve success? *Front. Psychol.* 14:1280783. 10.3389/fpsyg.2023.1280783 38145001 PMC10748581

[B42] Lyubomirsky S. King L. Diener E. (2005). The benefits of frequent positive affect: Does happiness lead to success? *Psychol. Bull.* 131 803–855. 10.1037/0033-2909.131.6.803 16351326

[B43] Ma L. Shahbaz P. Haq S. U. Boz I. (2023). Exploring the moderating role of environmental education in promoting a clean environment. *Sustainability* 15:8127. 10.3390/su15108127

[B44] Manavella A. Paoloni P. Rinaudo M. (2021). Compromiso con los aprendizajes: Perspectivas teóricas, antecedentes y reflexiones. *Ikastorratza eJ. Didact.* 27 30–60. 10.37261/27_alea/2

[B45] Melin M. (2012). *Once experienced, never ignored active learning as a tool for behavior change: A case study of world wide opportunities on organic farms.* Master thesis series in environmental studies and sustainability science, Lund University: Lund.

[B46] Meyer D. Turner J. (2006). Re-conceptualizing emotion and motivation to learn in classroom contexts. *Educ. Psychol. Rev.* 18 377–390. 10.1007/S10648-006-9032-1

[B47] Nainggolan A. (2024). The influence of environment and facilities on student learning in elementary schools. *Int. J. Stud. Educ.* 247–250. 10.62966/ijose.vi.773

[B48] Perry G. Richardson S. Harré N. Hodges D. Lyver P. Maseyk F. (2021). Evaluating the role of social norms in fostering pro-environmental behaviors. *Front. Environ. Sci.* 9:620125. 10.3389/fenvs.2021.620125

[B49] Philp J. Duchesne S. (2016). Exploring engagement in tasks in the language classroom. *Annu. Rev. Appl. Linguist.* 36 50–72. 10.1017/s0267190515000094

[B50] Pietarinen J. Soini T. Pyhältö K. (2014). Students’ emotional and cognitive engagement as the determinants of well-being and achievement in school. *Int. J. Educ. Res.* 67 40–51. 10.1016/J.IJER.2014.05.001

[B51] Pike G. Smart J. Ethington C. (2012). The mediating effects of student engagement on the relationships between academic disciplines and learning outcomes: An extension of Holland’s theory. *Res. High. Educ.* 53 550–575. 10.1007/S11162-011-9239-Y

[B52] Priya K. Thenmozhi S. (2021). Study on self-efficacy and pro-environmental behavior among school students. *Int. J. Indian Psychȯl.* 9 1851–1858. 10.25215/0902.184

[B53] Rahman M. Singh M. (2020). Language ideology of English-medium instruction in higher education. *English Today* 36 40–46. 10.1017/S0266078419000294

[B54] Ramkissoon H. Smith L. D. G. Weiler B. (2013). Relationships between place attachment, place satisfaction and pro-environmental behavior in an Australian national park. *J. Sustain. Tour.* 21 434–457. 10.1080/09669582.2012.708042

[B55] Ren Q. M. (2022). The formulation and verification of the scale of multidimensional evaluation for student engagement in college English classroom. *Shandong Foreign Lang. Teach.* 4 58–66.

[B56] Rogaten J. Rienties B. (2021). A critical review of learning gains methods and approaches. *Learn. Gain High. Educ.* 14 17–31. 10.1108/S1479-362820210000014003

[B57] Rüppel F. Liersch S. Walter U. (2015). The influence of psychological well-being on academic success. *J. Public Health* 23 15–24. 10.1007/s10389-015-0654-y

[B58] Skinner E. A. Pitzer J. R. (2012). “Developmental dynamics of student engagement, coping, and everyday resilience,” in *Handbook of research on student engagement*, eds Christenson S. Reschly A. Wylie C. (Boston, MA: Springer US), 21–44.

[B59] Steg L. de Groot J. (2010). Explaining prosocial intentions: Testing causal relationships in the norm activation model. *Br. J. Soc. Psychol.* 49 725–743. 10.1348/014466609X477745 20021707

[B60] Steinmayr R. Crede J. McElvany N. Wirthwein L. (2016). Subjective well-being, test anxiety, academic achievement: Testing for reciprocal effects. *Front. Psychol.* 6:1994. 10.3389/fpsyg.2015.01994 26779096 PMC4705295

[B61] Stern P. C. Dietz T. Abel T. Guagnano G. A. Kalof L. (1999). A value-belief-norm theory of support for social movements: The case of environmentalism. *Hum. Ecol. Rev.* 6 81–98.

[B62] Suárez-Varela M. Guardiola J. González-Gómez F. (2016). Do pro-environmental behaviors and awareness contribute to improve subjective well-being? *Appl. Res. Qual. Life* 11 429–444. 10.1007/S11482-014-9372-9

[B63] Suldo S. Thalji A. Ferron J. (2011). Longitudinal academic outcomes predicted by early adolescents’ subjective well-being, psychopathology, and mental health status yielded from a dual factor model. *J. Posit. Psychol.* 6 17–30. 10.1080/17439760.2010.536774

[B64] Sulis G. (2023). Exploring learner engagement with languages (LX) within and beyond the English classroom. *Lang. Teach. Res.* 14:13621688231216869. 10.1177/13621688231216869 PMC13347788 42428573

[B65] Sulphey M. M. Faisal S. (2021). Connectedness to nature and environmental concern as antecedents of commitment to environmental sustainability. *Int. J. Energy Econ. Policy* 11 208–219.

[B66] Sun Y. Liu N. Zhao M. (2019). Factors and mechanisms affecting green consumption in China: A multilevel analysis. *J. Clean. Prod.* 209 481–493. 10.1016/J.JCLEPRO.2018.10.241

[B67] Wang M. Degol J. Henry D. (2019). An integrative development-in-sociocultural-context model for children’s engagement in learning. *Am. Psychol.* 749 1086–1102. 10.1037/amp0000522 31829690

[B68] Williams D. R. Vaske J. J. (2003). The measurement of place attachment: Validity and generalizability of a psychometric approach. *For. Sci.* 49 830–840. 10.1093/forestscience/49.6.830

[B69] Wu S. Cheng X. Qiao J. (2023). The impact of proactive personality on pro-environmental behavior: The mediating role of subjective well-being. *J. Huainan Norm. Univ.* 25 124–128.

[B70] Xie X. (2002). Language learning strategies and foreign language teaching. *J. Yangzhou Univ.* 4, 89–91.

[B71] Xing Z. J. (2002). To develop an inventory for measuring the subjective well-being of Chinese citizens. *Hong Kong J. Soc. Sci.* 23 151–189.

[B72] Yang Y. N. (2024). *The influence of awe on pro-environmental behavior of middle school students: The role of situational publicity and social norms.* Master’s thesis, Shanxi University: Taiyuan. 10.27284/d.cnki.gsxiu.2024.001142

[B73] Yang Y. Zhou C. Wang Z. (2024). The relationship between self-control and learning engagement among Chinese college students: The chain mediating roles of resilience and positive emotions. *Front. Psychol.* 15:1331691. 10.3389/fpsyg.2024.1331691 38445063 PMC10913274

[B74] Zawadzki S. Steg L. Bouman T. (2020). Meta-analytic evidence for a robust and positive association between individuals’ pro-environmental behaviors and their subjective wellbeing. *Environ. Res. Lett.* 15:123007. 10.1088/1748-9326/abc4ae

[B75] Zelenski J. Desrochers J. (2021). Can positive and self-transcendent emotions promote pro-environmental behavior? *Curr. Opin. Psychol.* 42 31–35. 10.1016/j.copsyc.2021.02.009 33819735

[B76] Zhou H. Long L. (2004). Statistical Remedies for Common Method Biases. *Adv. Psychol. Sci.* 12 942–950.

[B77] Zhou L. (2006). Increase the language input and improve the language output. *J. Nanping Teach. Coll.* 25, 92–95. 10.3969/j.issn.1674-2109.2006.03.026

[B78] Zilvinskis J. Masseria A. A. Pike G. (2017). Student engagement and student learning: Examining the convergent and discriminant validity of the revised national survey of student engagement. *Res. High. Educ.* 58 880–903. 10.1007/s11162-017-9450-6

[B79] Zimmerman B. (2000). Self-efficacy: An essential motive to learn. *Contemp. Educ. Psychol.* 25 82–91. 10.1006/ceps.1999.1016 10620383

